# Supplementary material for: Therapeutic Potential of Ramalin Derivatives with Enhanced Stability in the Treatment of Alzheimer’s Disease
Source: Molecules. 2024 Nov 5;29(22):5223. doi: 10.3390/molecules29225223 (PMC11597085; doi:10.3390/molecules29225223)
Supplement: Supplementary file 1 [file molecules-29-05223-s001.zip › molecules-3261724-supplementary.pdf]

molecules

Supporting information

Therapeutic Potential of Ramalin Derivatives with Enhanced Stability in the Treatment of Alzheimer's Disease

Tai Kyoung Kim [1,†], Ju-Mi Hong [1,†], Jaewon Kim [1,2], Kyung Hee Kim [1,3], Se Jong Han [1], Il-Chan Kim [1], Hyuncheol Oh [4], Dong-Gyu Jo [5,*] and Joung Han Yim [1,*]

[1] Division of Polar Life Sciences, Korea Polar Research Institute, Incheon 21990, Republic of Korea; tkkim@kopri.re.kr (T.K.K.); wnal5555@kopri.re.kr (J.-M.H.); ashcercle@kopri.re.kr (J.K.); kh313@kopri.re.kr (K.H.K.); hansj@kopri.re.kr (S.J.H.); ickim@kopri.re.kr (I.-C.K.)
[2] Department of Plant Biotechnology, Korea University, Seoul 02841, Republic of Korea
[3] Department of Chemistry, Hanseo University, Seosan 31962, Republic of Korea
[4] College of Pharmacy, Wonkwang University, Iksan 54538, Republic of Korea; hoh@wku.ac.kr
[5] School of Pharmacy, Sungkyunkwan University, Suwon 16419, Republic of Korea
* Correspondence: jodg@skku.edu (D.-G.J.); jhyim@kopri.re.kr (J.H.Y.); Tel.: +82-31-290-7776 (D.-G.J.); +82-32-760-5540 (J.H.Y.); Fax: +82-32-760-5509 (J.H.Y.)
† These authors contributed equally to this work.

Context ....page

N^5-(methyl(phenyl)amino)-L-glutamine (**RA-Hyd-Me**)

Figure S1. ^{1}H NMR spectrum of **RA-Hyd-Me** 4

Figure S2. ^{13}C NMR spectrum of **RA-Hyd-Me** 4

Figure S3. DEPT spectrum of **RA-Hyd-Me** 5

Figure S4. COSY spectrum of **RA-Hyd-Me** 5

Figure S5. HSQC spectrum of **RA-Hyd-Me** 6

Figure S6. HMBC spectrum of **RA-Hyd-Me** 6

Figure S7. HRESIMS spectrum of **RA-Hyd-Me** 7

Figure S8. FT-IR spectrum of **RA-Hyd-Me** 7

N^5-(methyl(m-tolyl)amino)-L-glutamine (**RA-Hyd-Me-Tol**)

Figure S9. ^{1}H NMR spectrum of **RA-Hyd-Me-Tol** 8

Figure S10. ^{13}C NMR spectrum of **RA-Hyd-Me-Tol** 8

Figure S11. DEPT spectrum of **RA-Hyd-Me-Tol** 9

Figure S12. COSY spectrum of **RA-Hyd-Me-Tol** 9

Figure S13. HSQC spectrum of **RA-Hyd-Me-Tol** 10

Figure S14. HMBC spectrum of **RA-Hyd-Me-Tol** 10

Figure S15. HRESIMS spectrum of **RA-Hyd-Me-Tol** 11

Figure S16. FT-IR spectrum of **RA-Hyd-Me-Tol** 11

N^5-benzamido-L-glutamine (**RA-Benzo**)

Figure S17. ^{1}H NMR spectrum of **RA-Benzo** 12

Figure S18. ^{13}C NMR spectrum of **RA-Benzo** 12

Figure S19. DEPT spectrum of **RA-Benzo** 13

Figure S20. COSY spectrum of **RA-Benzo** 13

Figure S21. HSQC spectrum of **RA-Benzo** 14

Figure S22. HMBC spectrum of **RA-Benzo** 14

Figure S23. HRESIMS spectrum of **RA-Benzo** 15

Figure S24. FT-IR spectrum of **RA-Benzo** 15

N^5-(2-hydroxybenzamido)-L-glutamine (**RA-Sali**)

Figure S25. ^{1}H NMR spectrum of **RA-Sali** 16

Figure S26. ^{13}C NMR spectrum of **RA-Sali** 16

Figure S27. DEPT spectrum of **RA-Sali** 17

Figure S28. COSY spectrum of **RA-Sali** 17

Figure S29. HSQC spectrum of **RA-Sali** 18

Figure S30. HMBC spectrum of **RA-Sali** 18

Figure S31. HRESIMS spectrum of **RA-Sali** 19

Figure S32. FT-IR spectrum of **RA-Sali** 19

Figure S33. Mass product ion scan spectrum of **Ramalin** 20

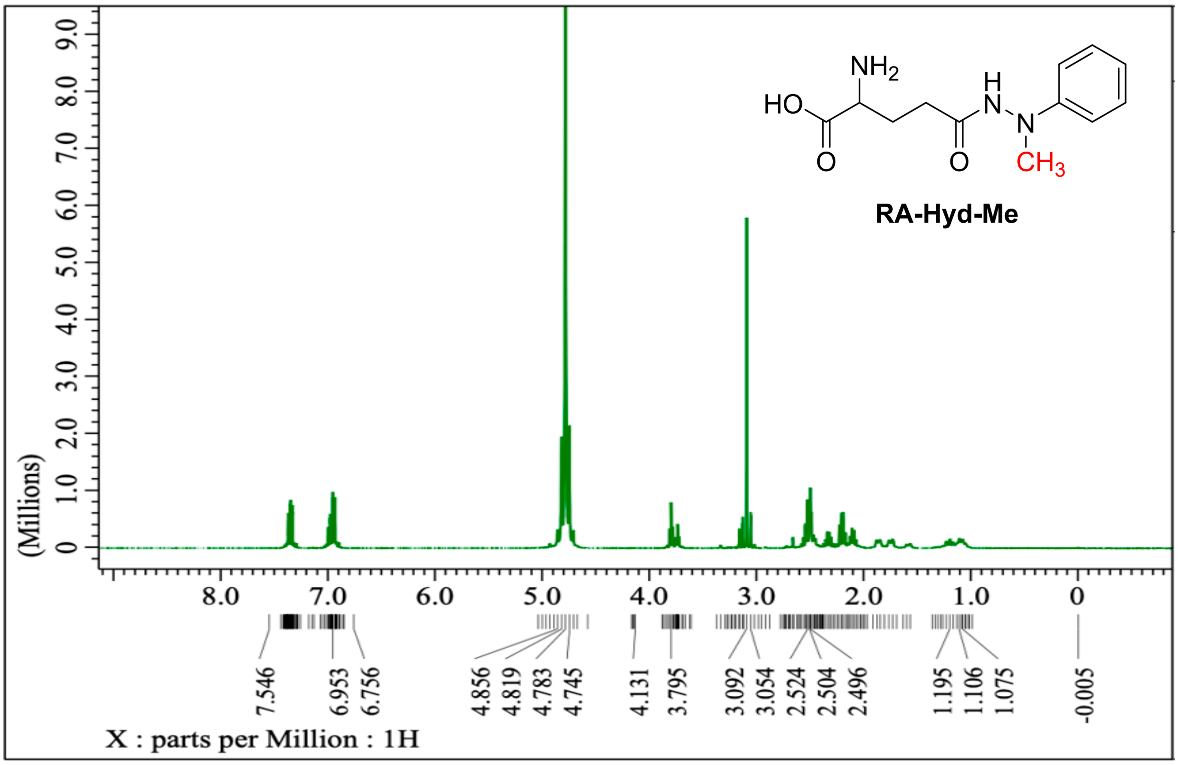

Figure S1: ^{1}H NMR (400 MHz) spectrum of **RA-Hyd-Me**

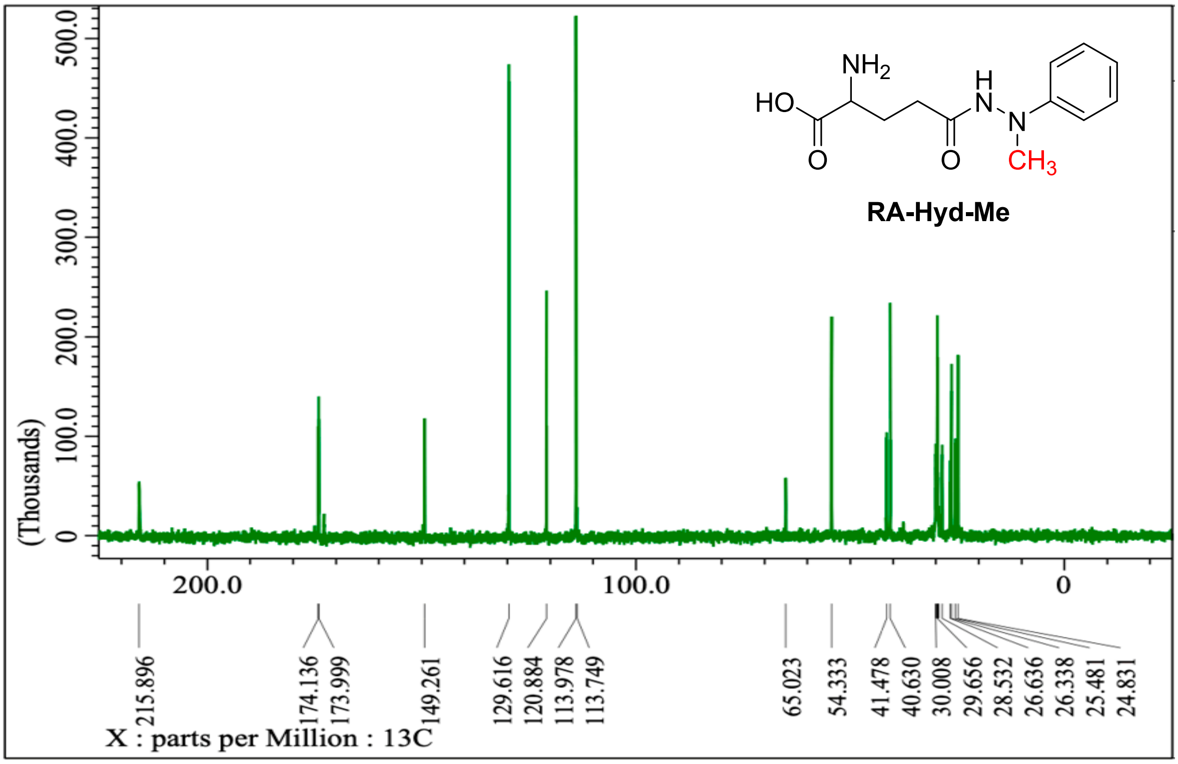

Figure S2: ^{13}C NMR (100 MHz) spectrum of **RA-Hyd-Me**

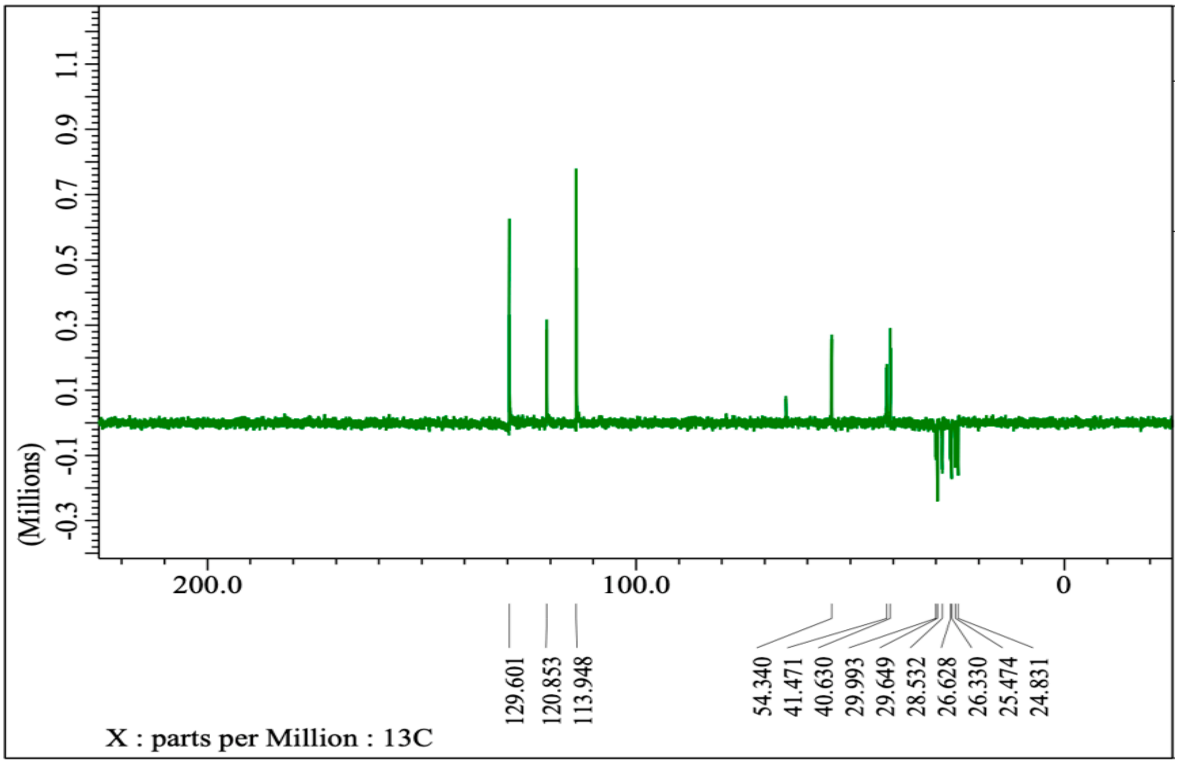

Figure S3: DEPT spectrum of **RA-Hyd-Me**

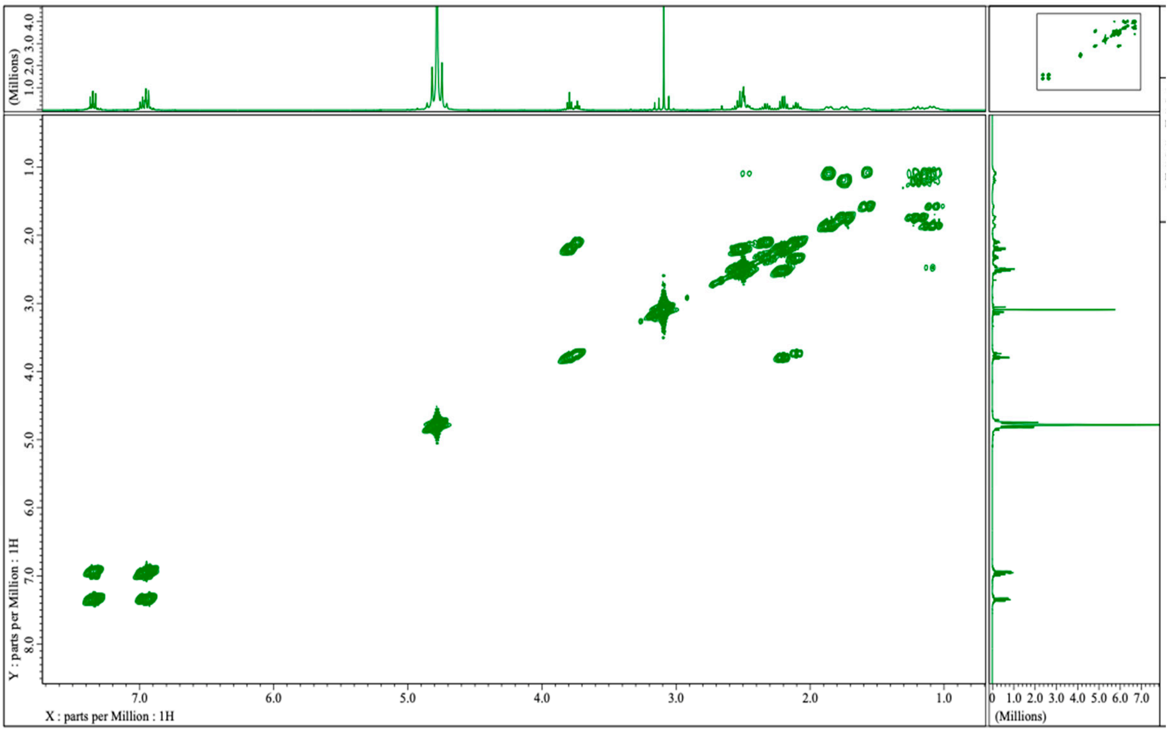

Figure S4: COSY spectrum of **RA-Hyd-Me**

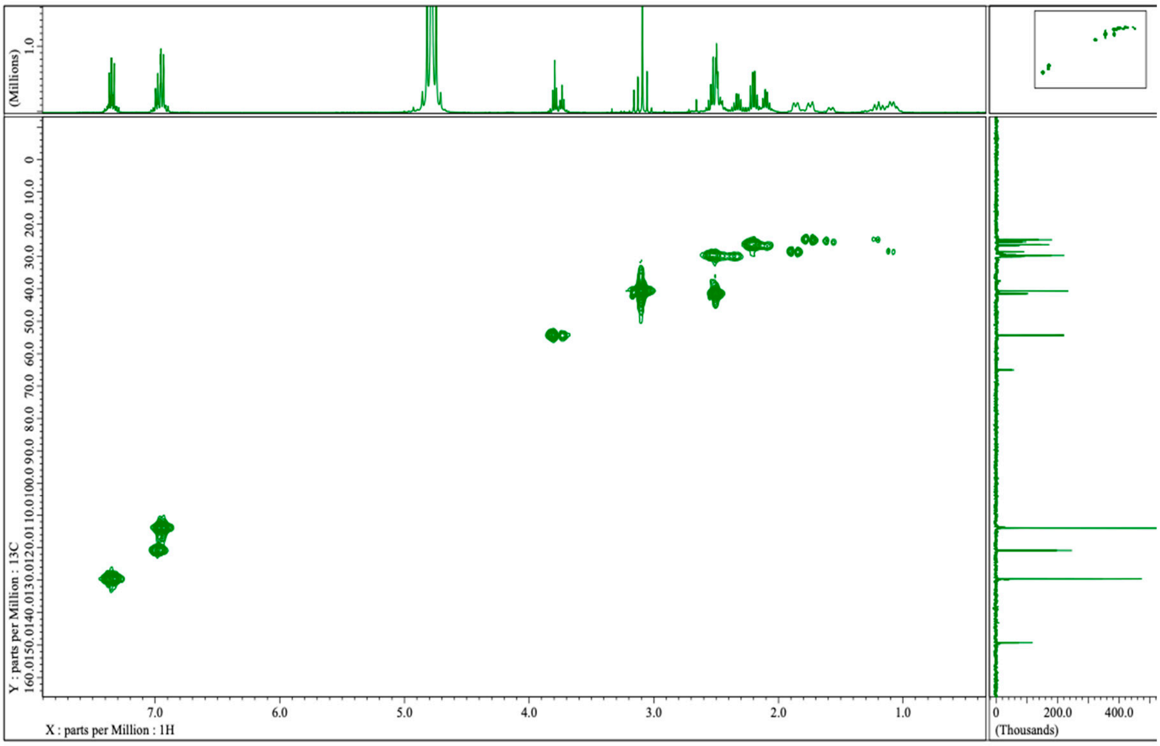

Figure S5: HMQC spectrum of **RA-Hyd-Me**

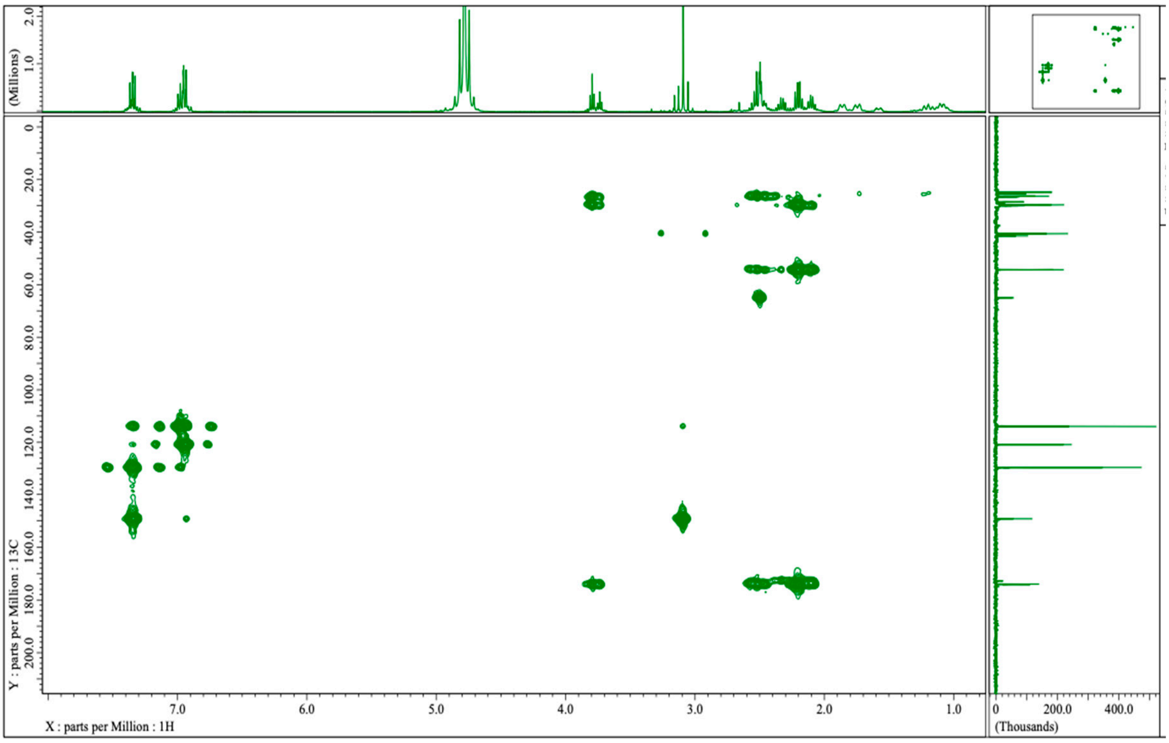

Figure S6: HMBC spectrum of **RA-Hyd-Me**

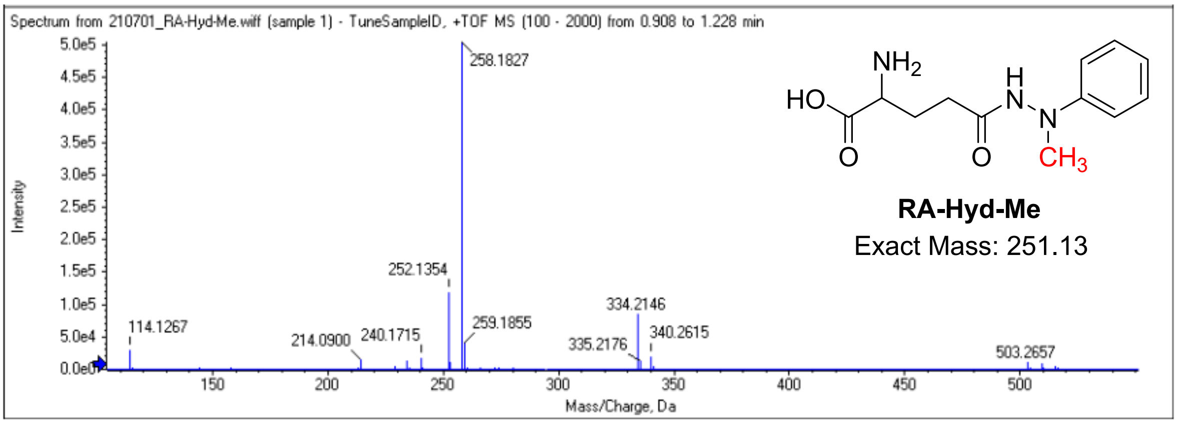

Figure S7: HRESIMS spectrum of **RA-Hyd-Me**

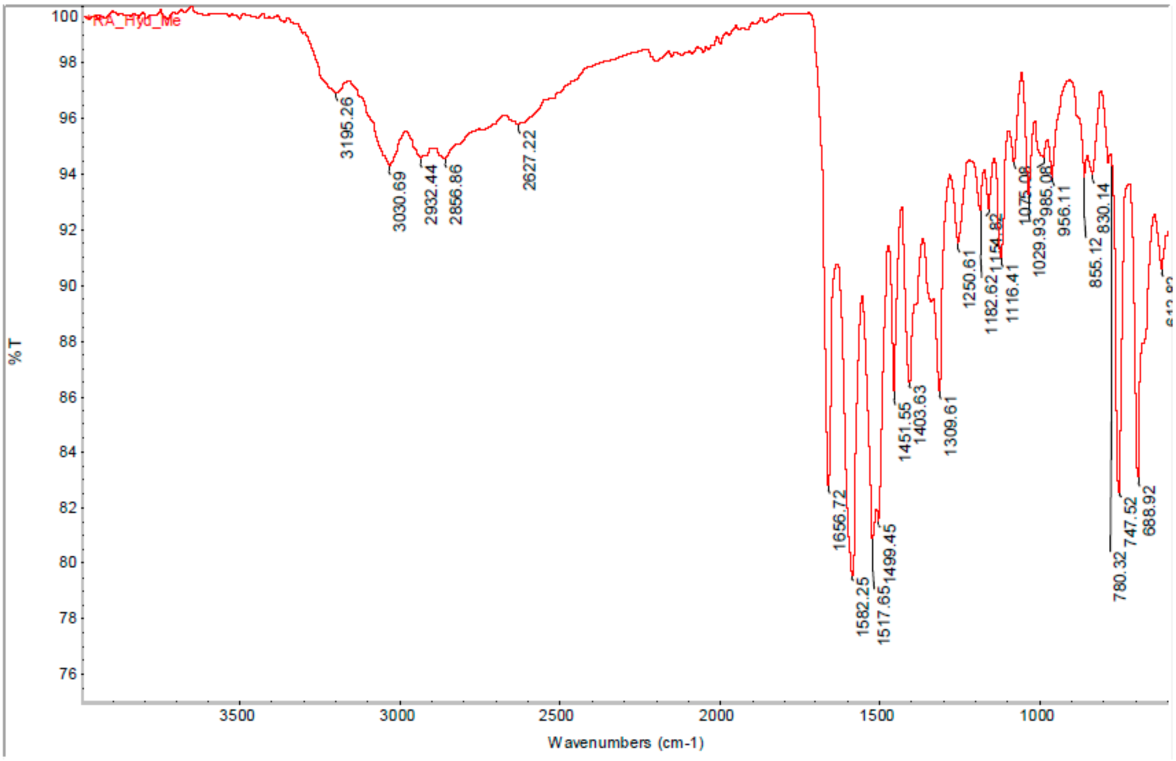

Figure S8: FT-IR spectrum of **RA-Hyd-Me**

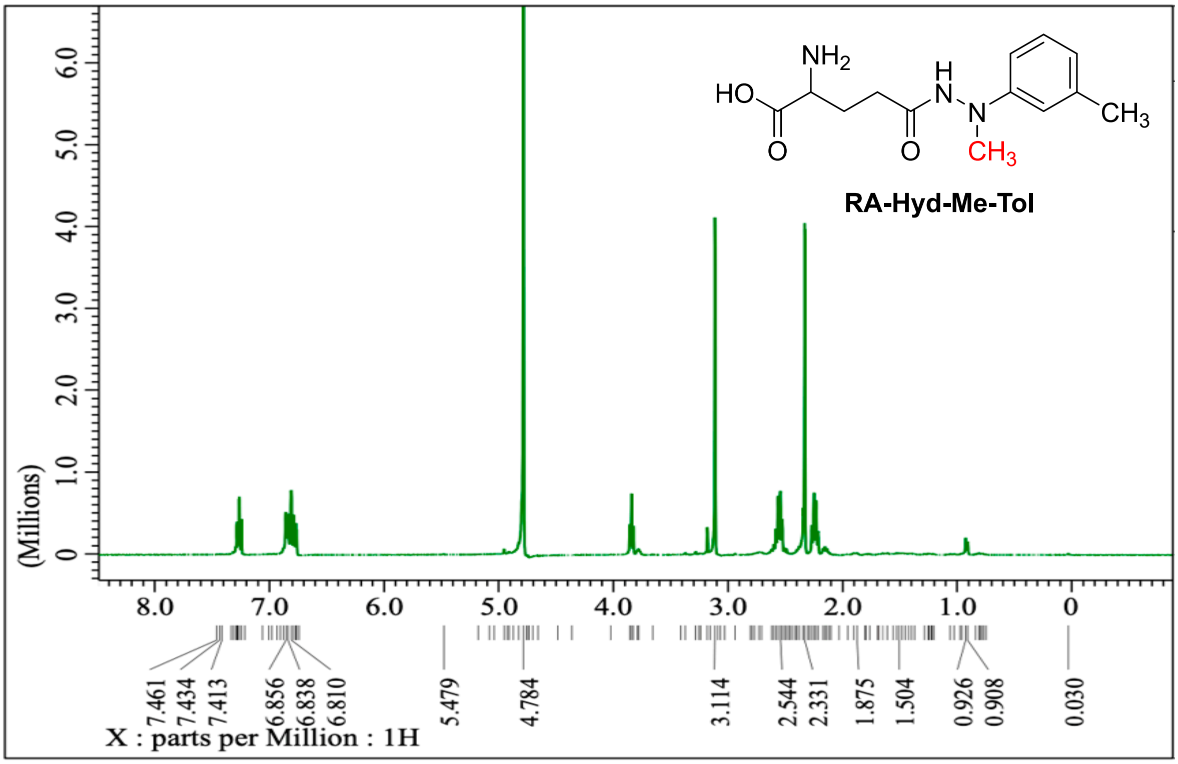

Figure S9: ^{1}H NMR (400 MHz) spectrum of **RA-Hyd-Me-Tol**

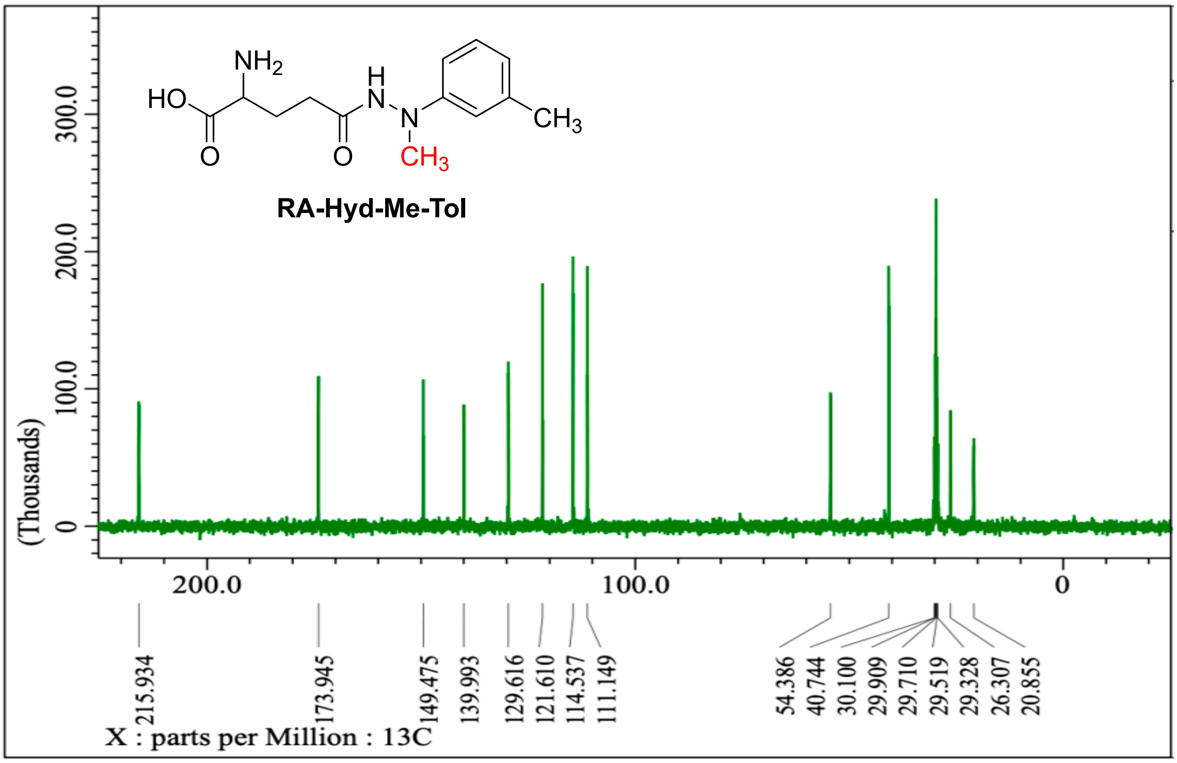

Figure S10: ^{13}C NMR (100 MHz) spectrum of **RA-Hyd-Me-Tol**

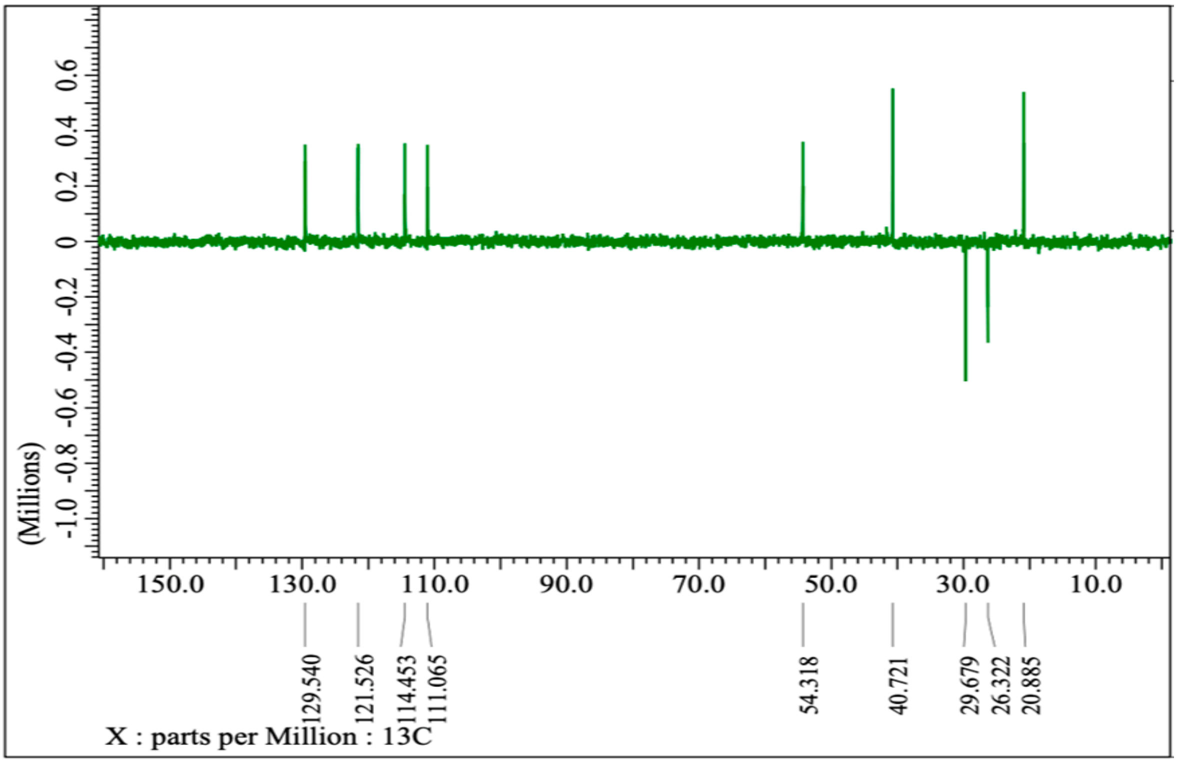

Figure S11: DEPT spectrum of **RA-Hyd-Me-Tol**

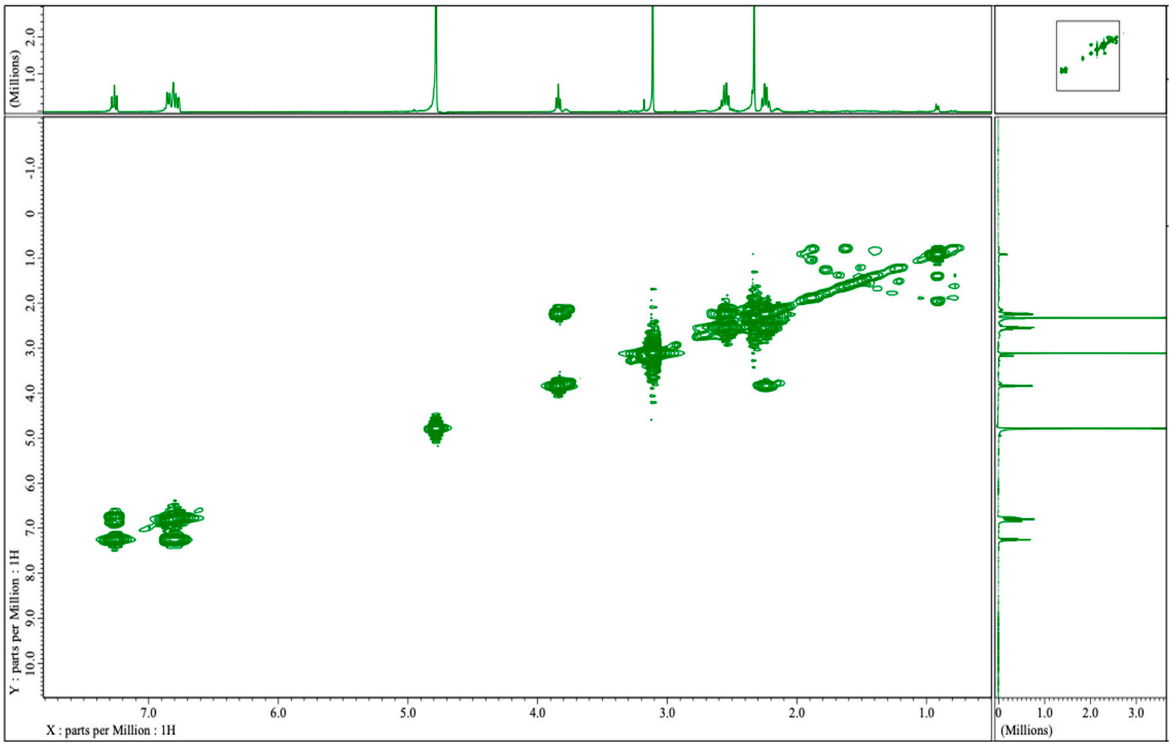

Figure S12: COSY spectrum of **RA-Hyd-Me-Tol**

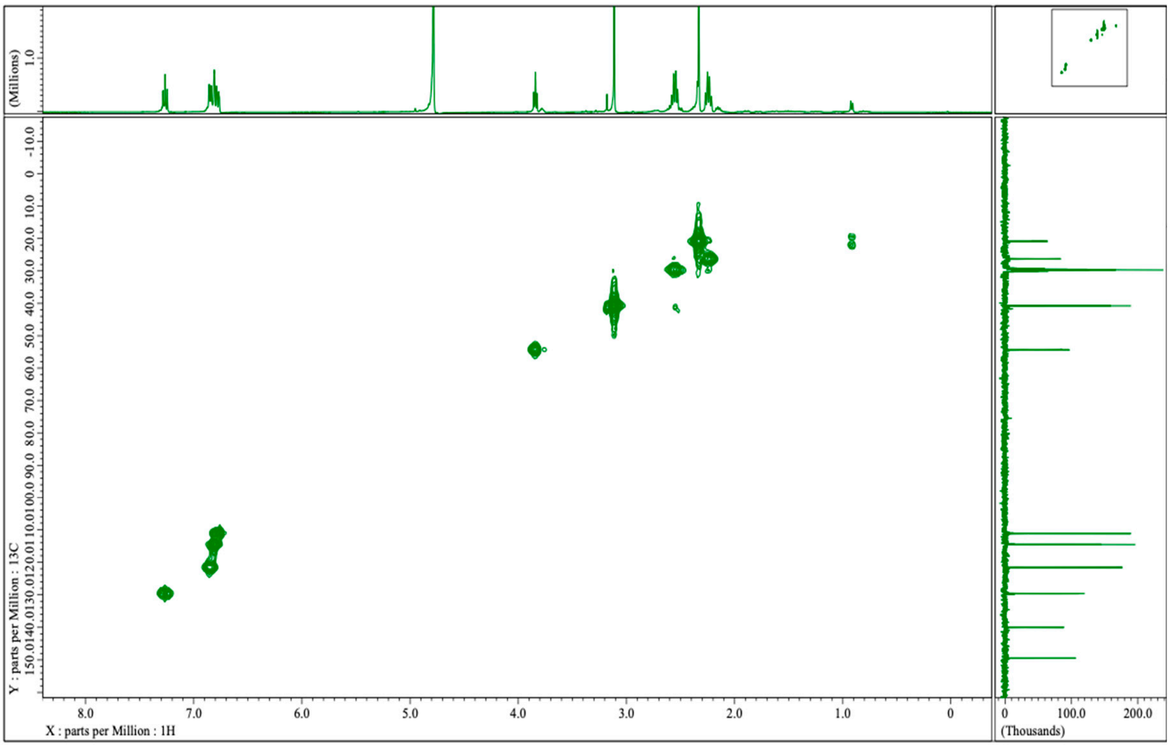

Figure S13: HMQC spectrum of **RA-Hyd-Me-Tol**

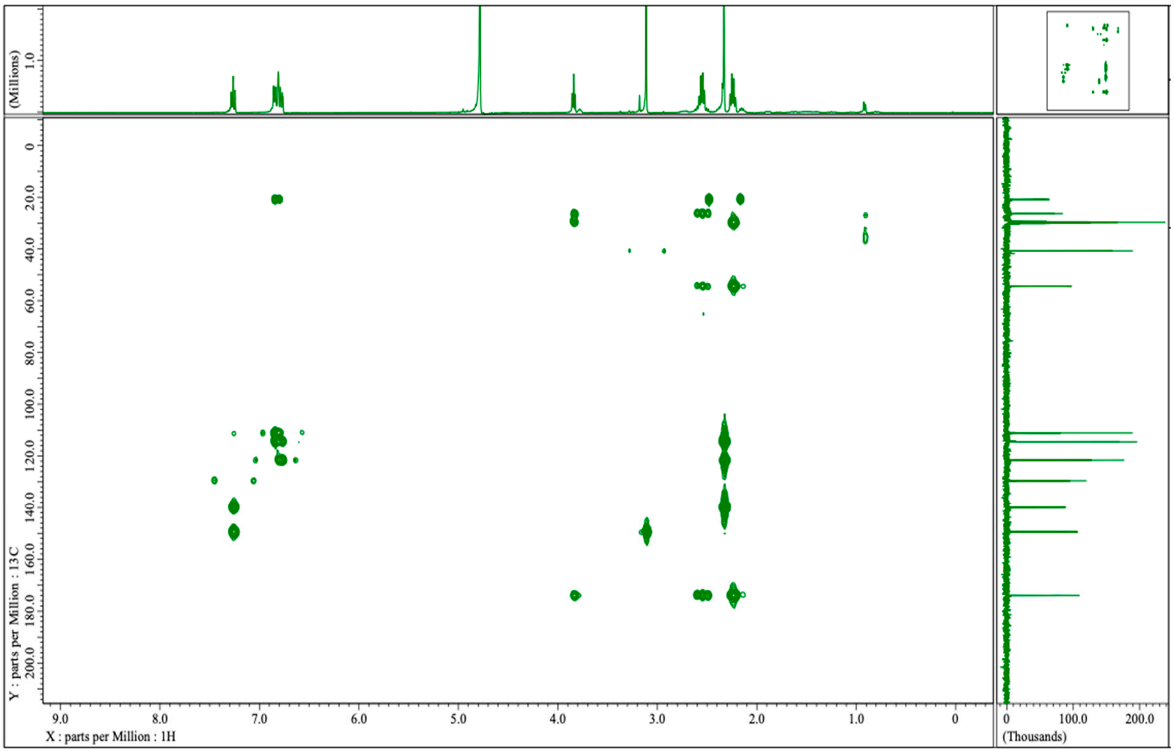

Figure S14: HMBC spectrum of **RA-Hyd-Me-Tol**

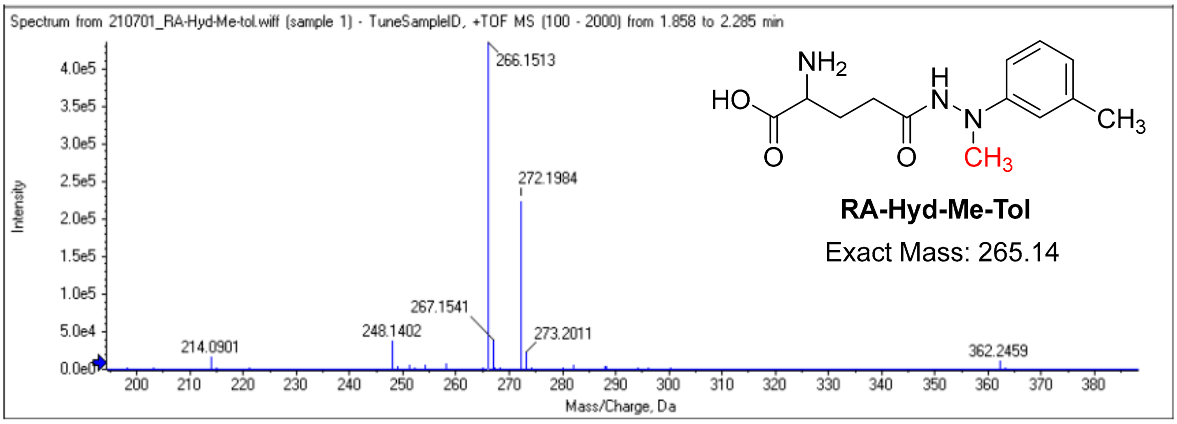

Figure S15: HRESIMS spectrum of **RA-Hyd-Me-Tol**

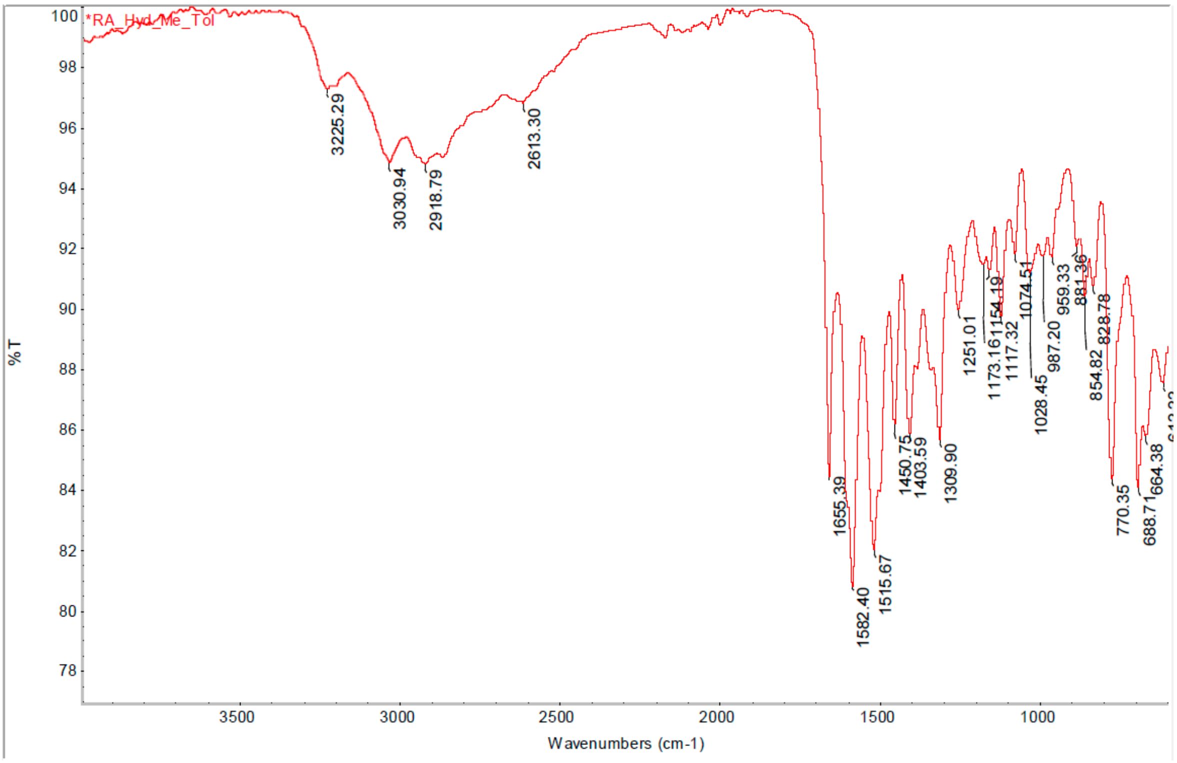

Figure S16: FT-IR spectrum of **RA-Hyd-Me-Tol**

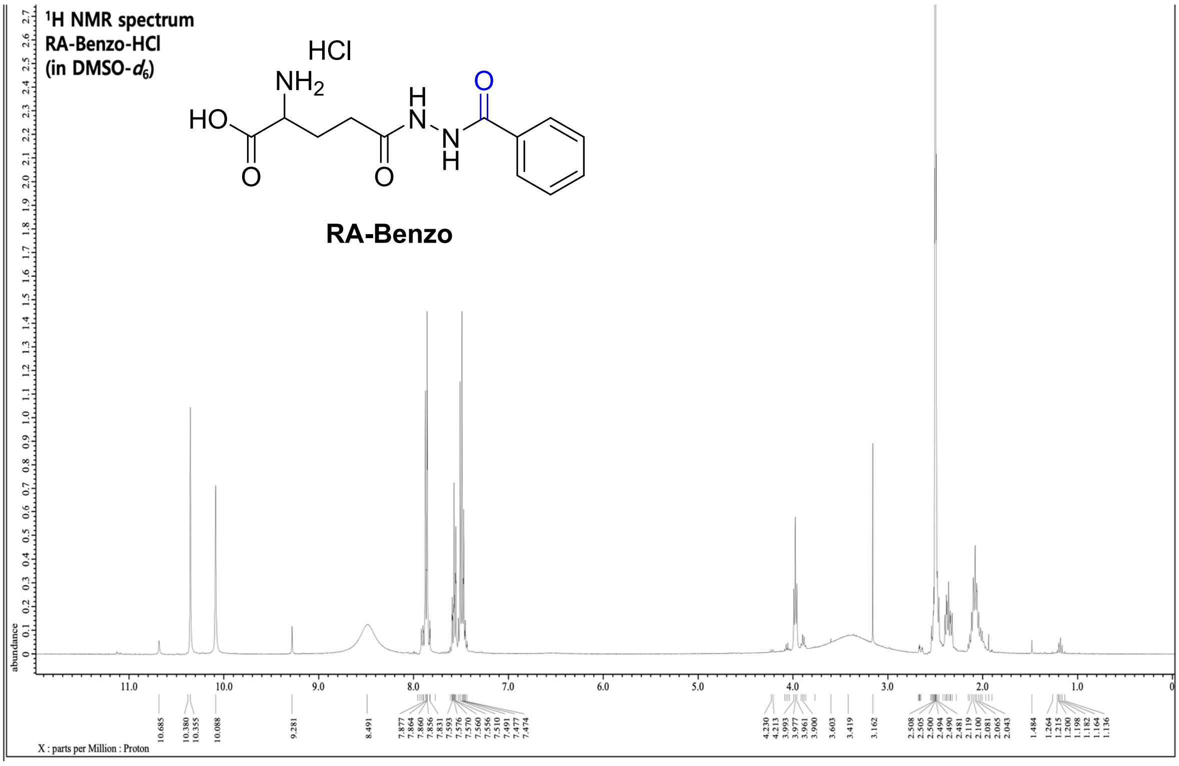

Figure S17: ^{1}H NMR (400 MHz) spectrum of **RA-Benzo**

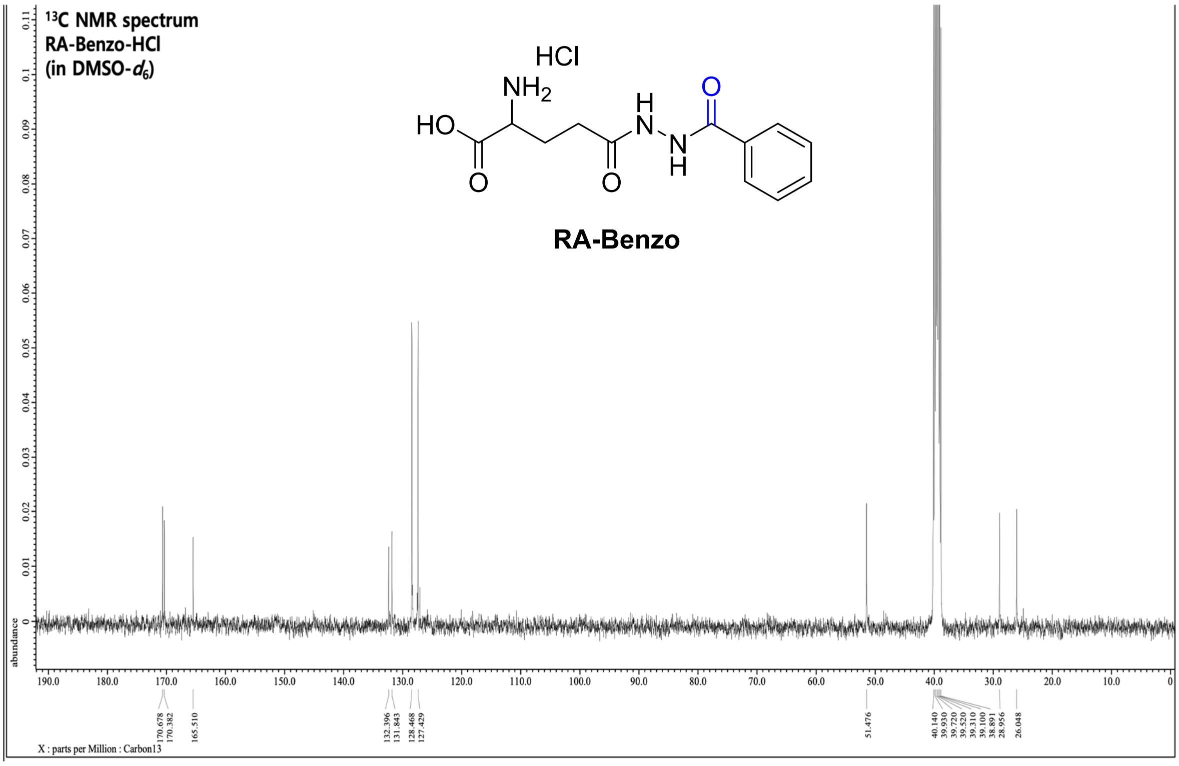

Figure S18: ^{13}C NMR (100 MHz) spectrum of **RA-Benzo**

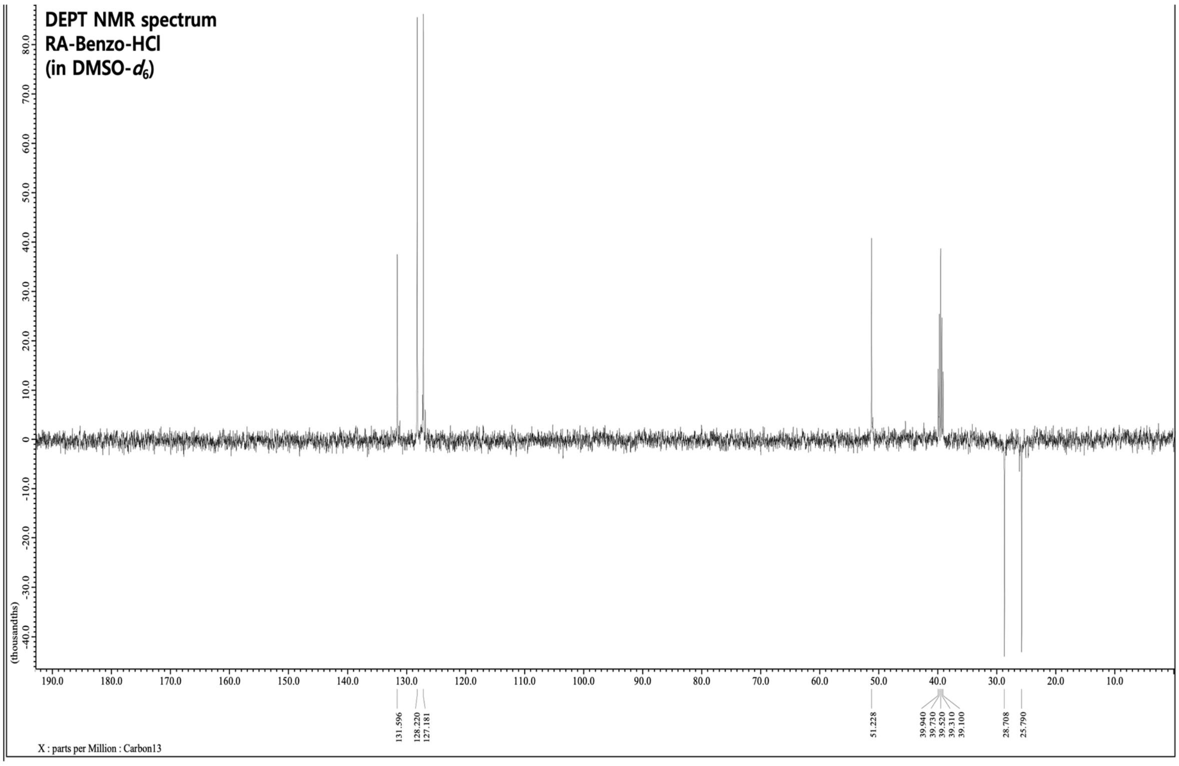

Figure S19: DEPT spectrum of **RA-Benzo**

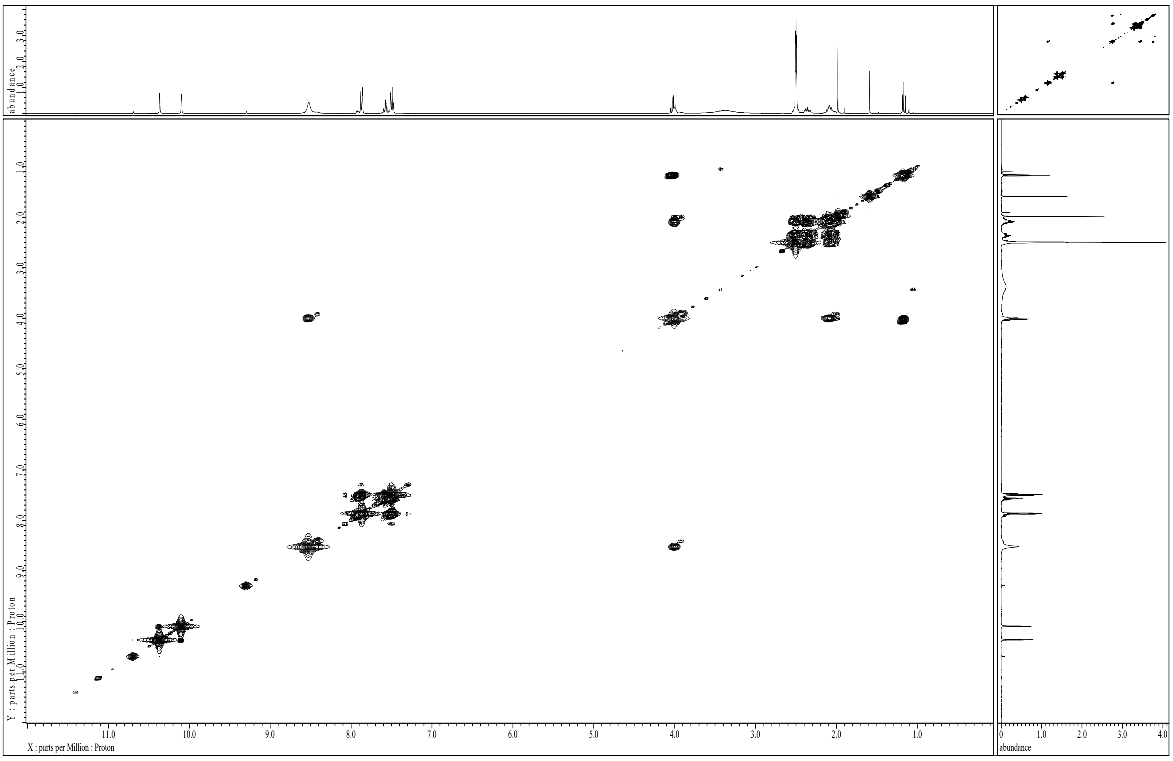

Figure S20: COSY spectrum of **RA-Benzo**

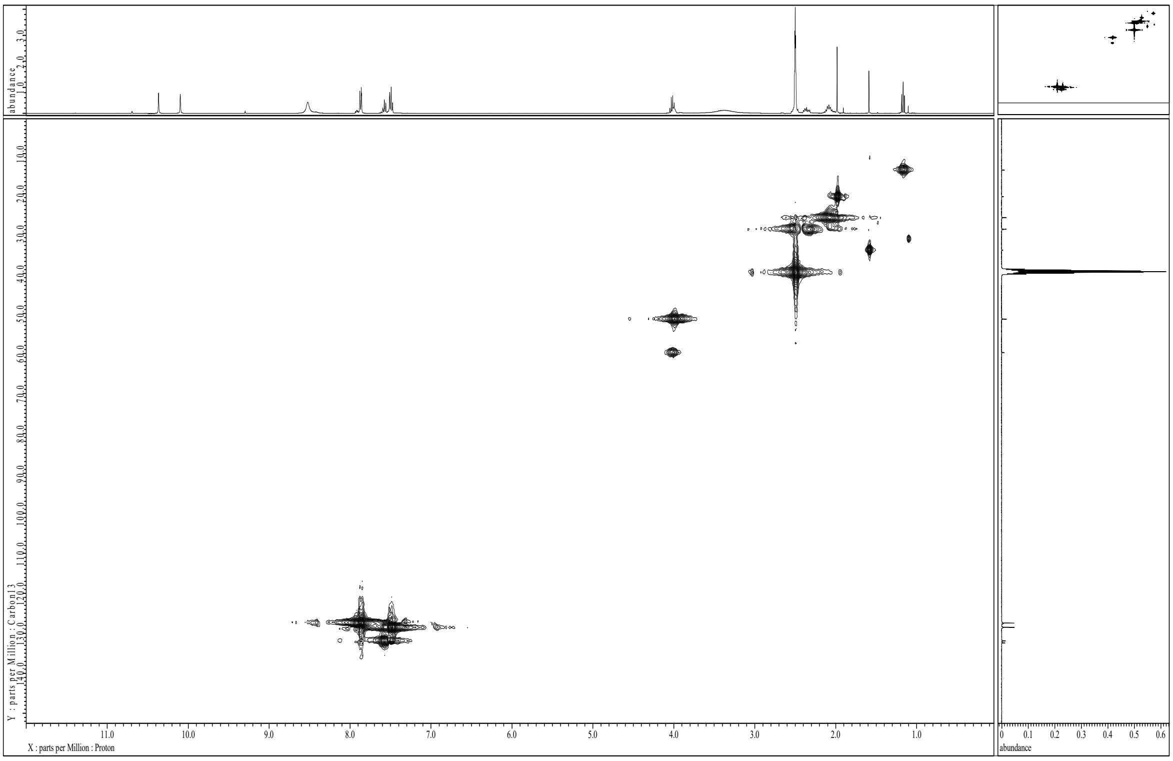

Figure S21: HMQC spectrum of **RA-Benzo**

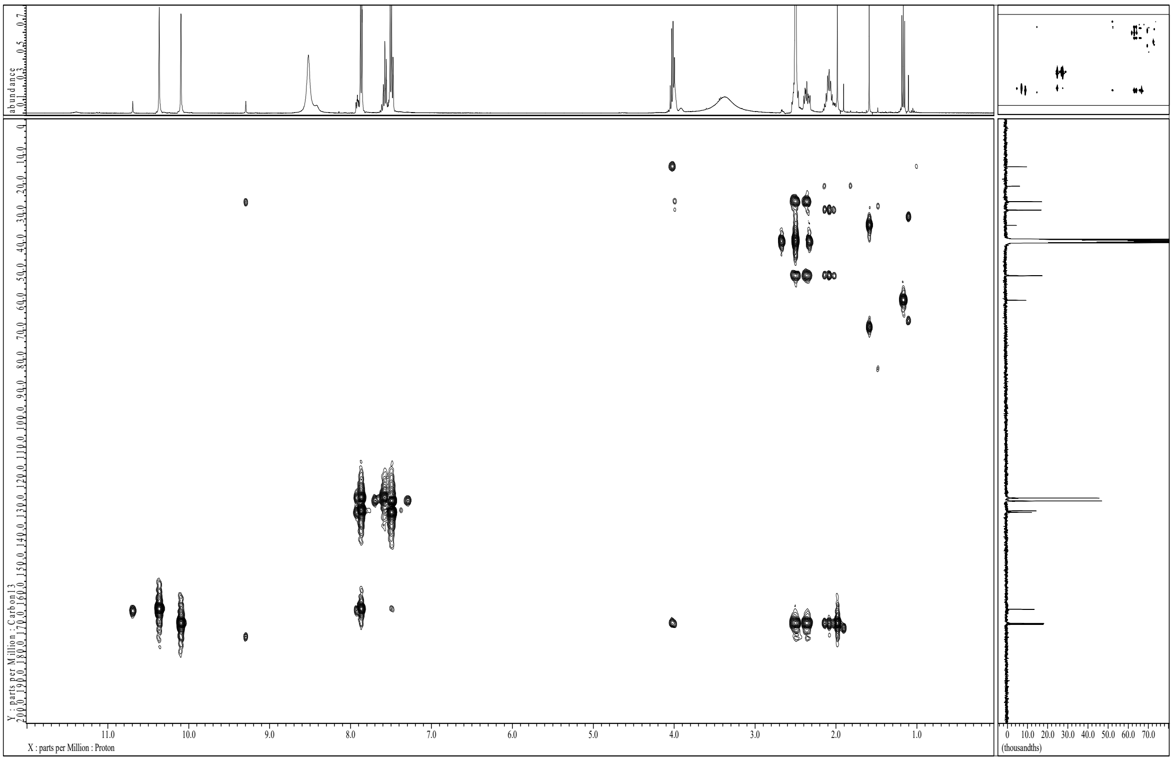

Figure S22: HMBC spectrum of **RA-Benzo**

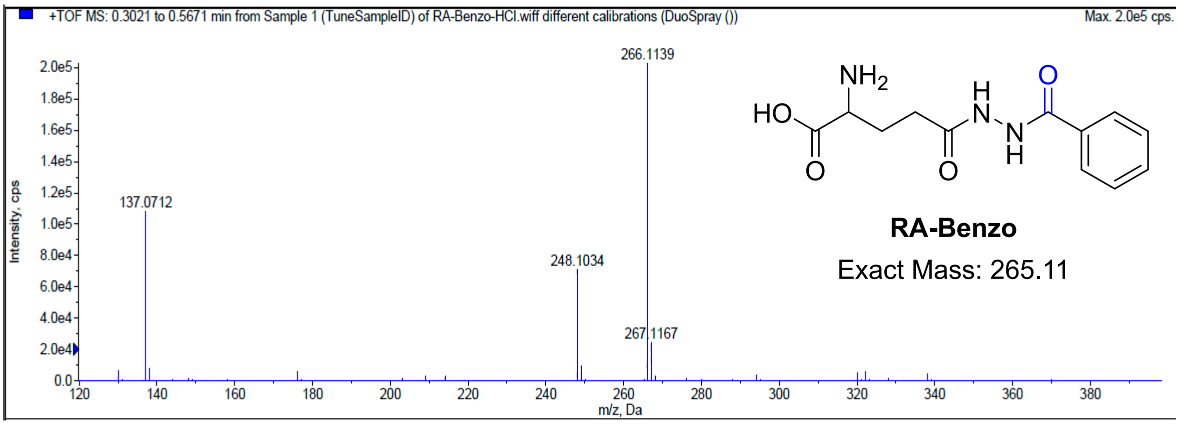

Figure S23: HRESIMS spectrum of **RA-Benzo**

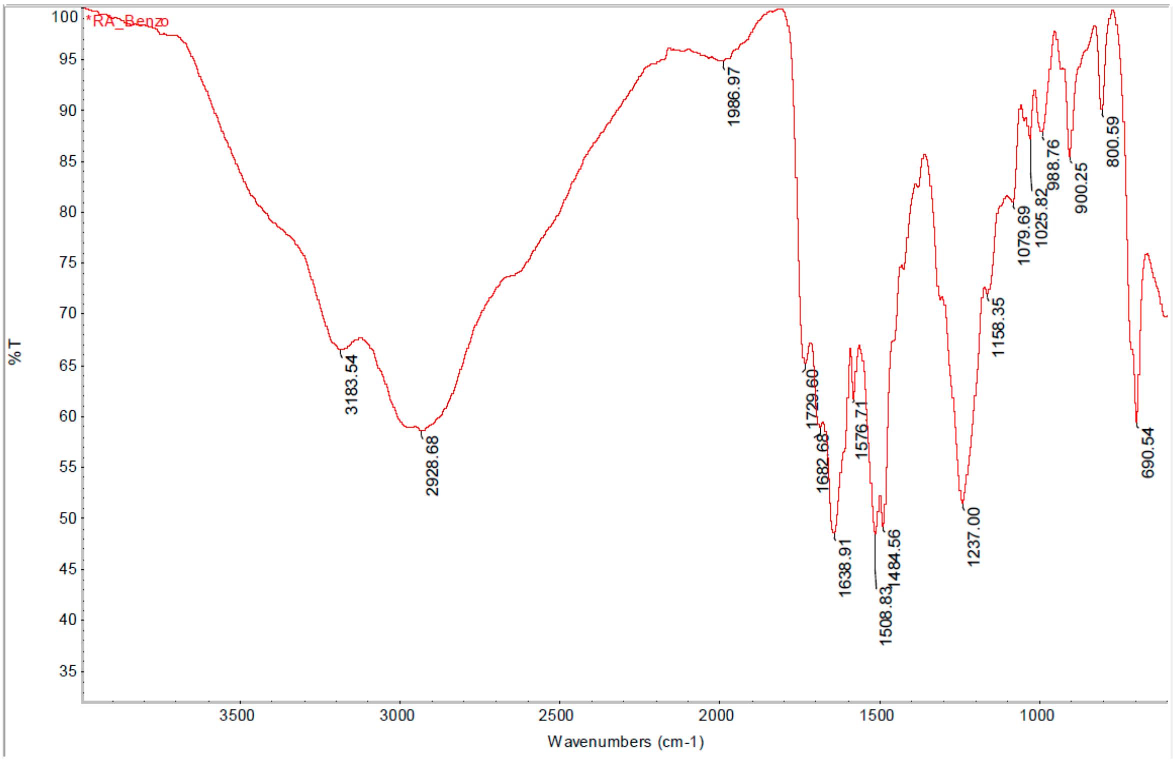

Figure S24: FT-IR spectrum of **RA-Benzo**

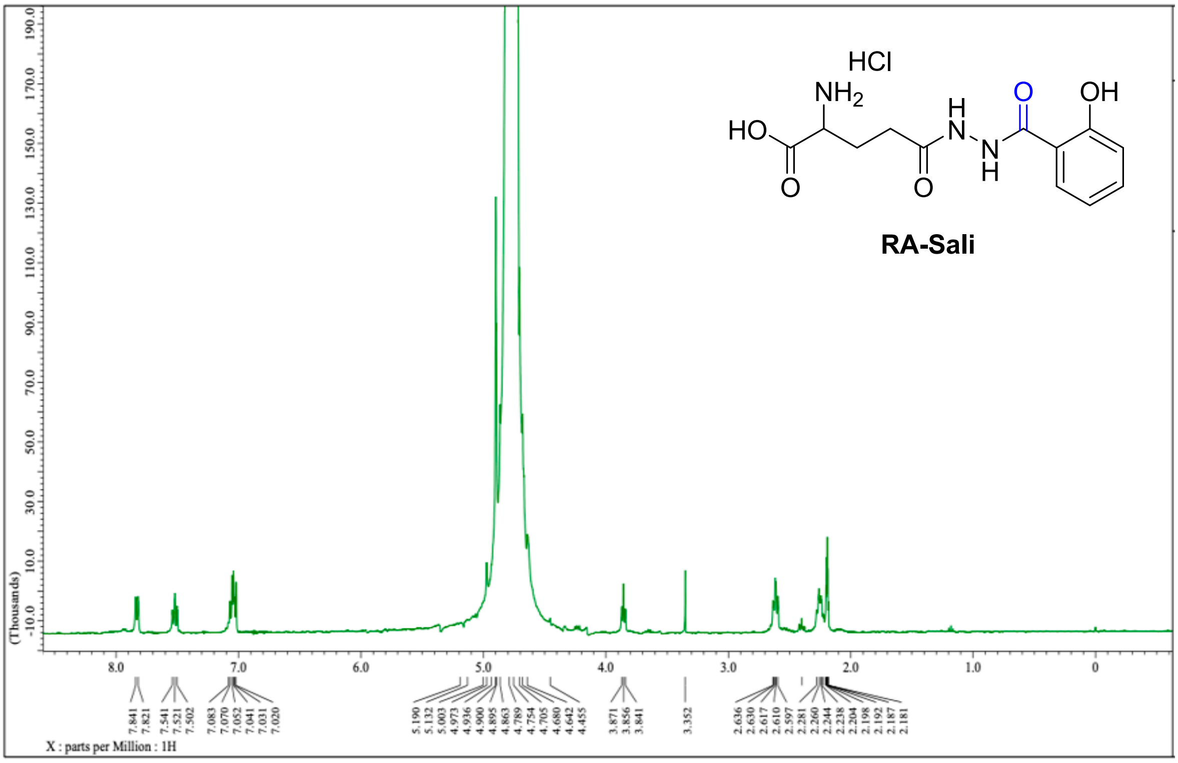

Figure S25: ^{1}H NMR (400 MHz) spectrum of **RA-Sali**

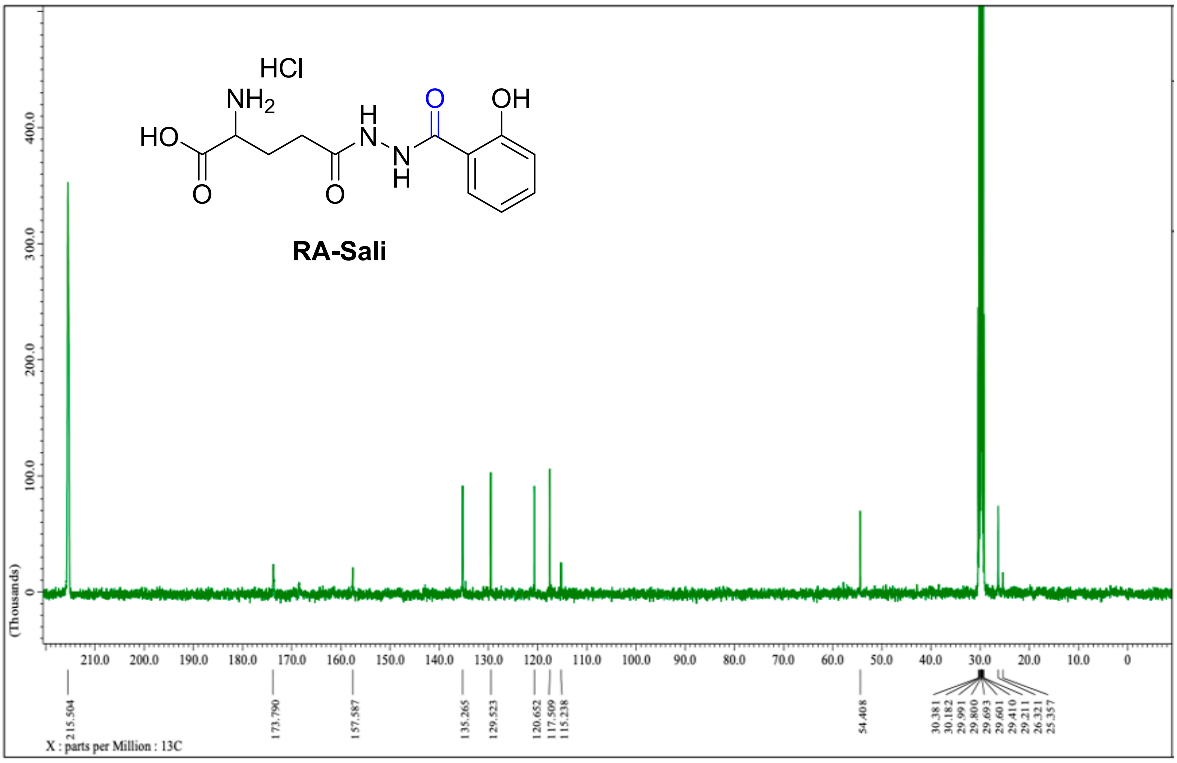

Figure S26: ^{13}C NMR (100 MHz) spectrum of **RA-Sali**

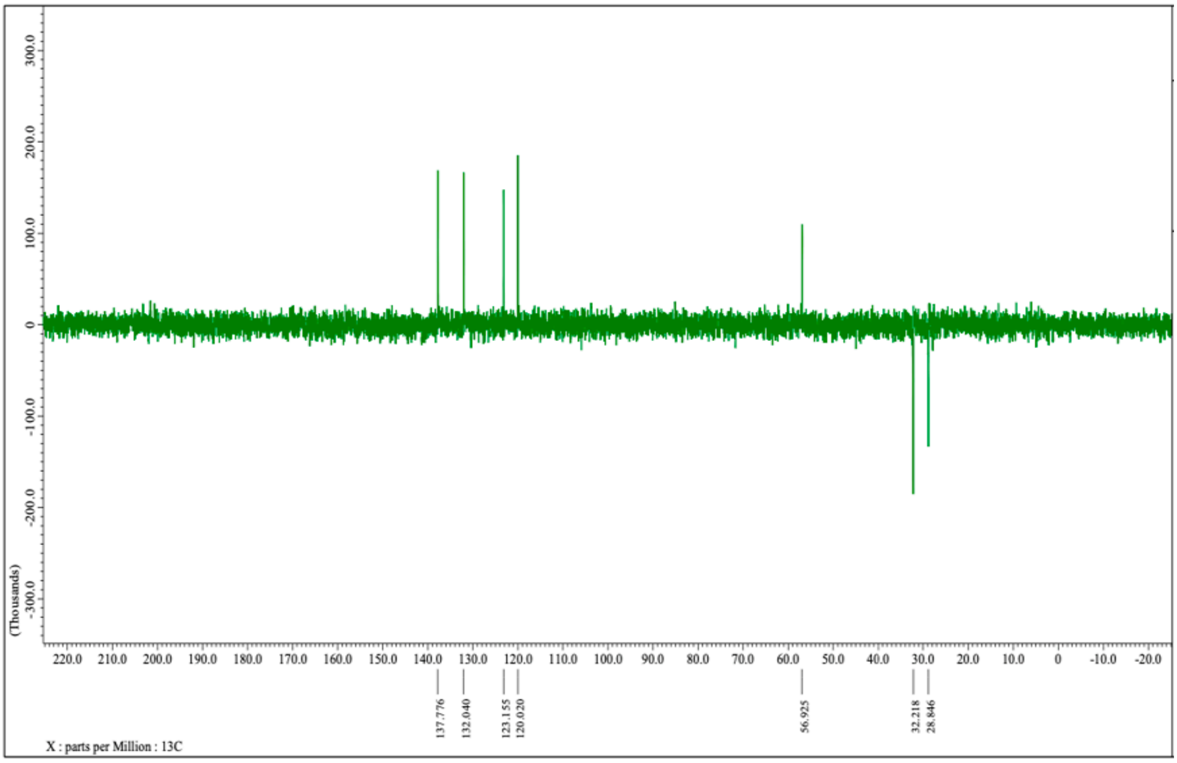

Figure S27: DEPT spectrum of **RA-Sali**

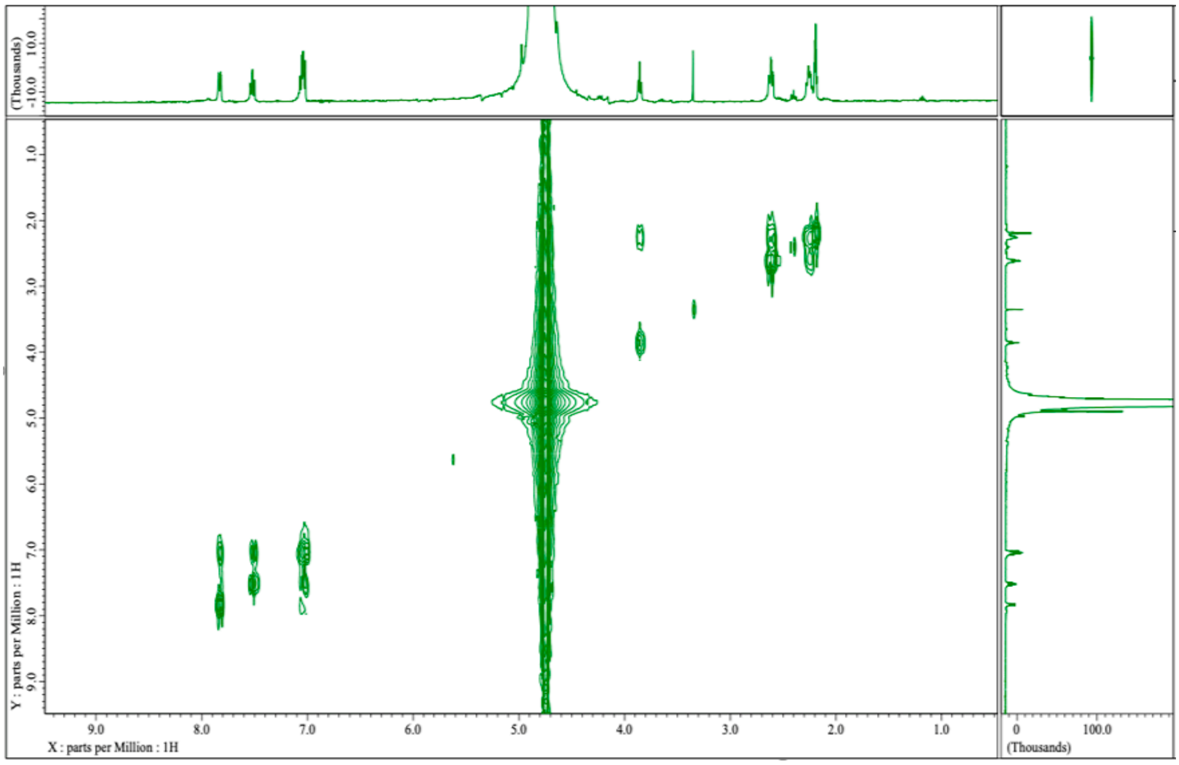

Figure S28: COSY spectrum of **RA-Sali**

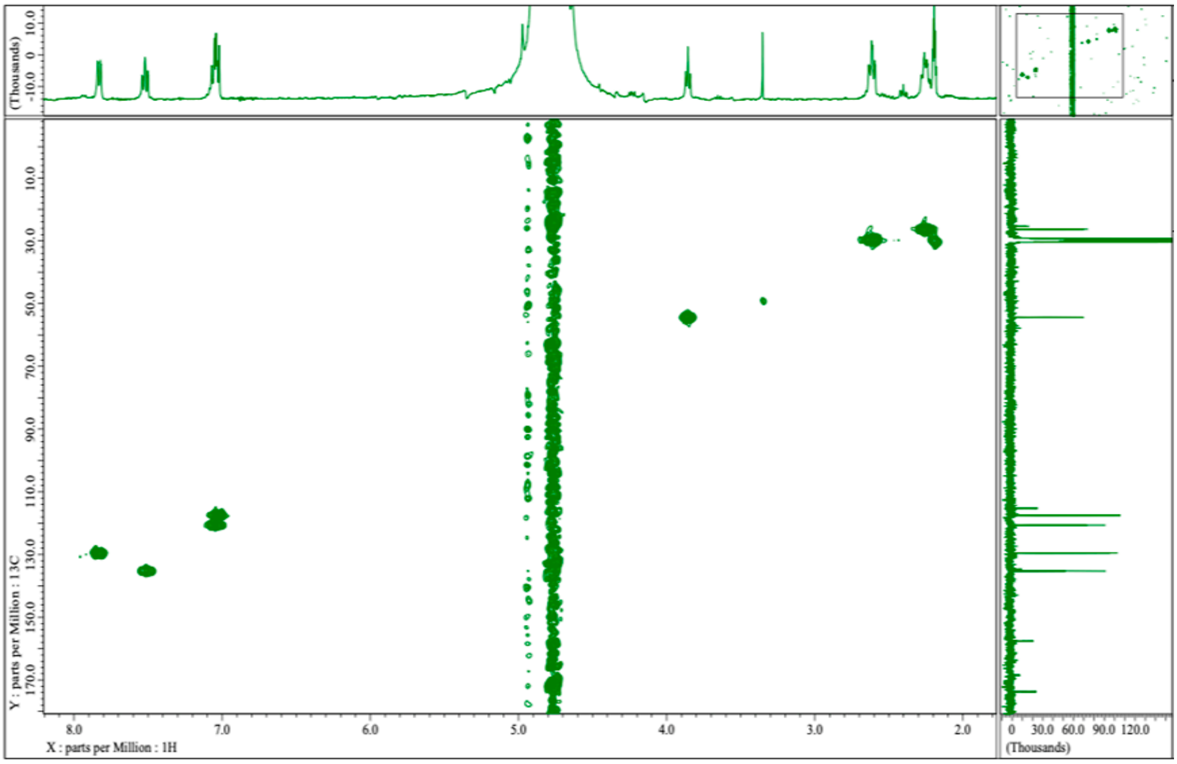

Figure S29: HMQC spectrum of **RA-Sali**

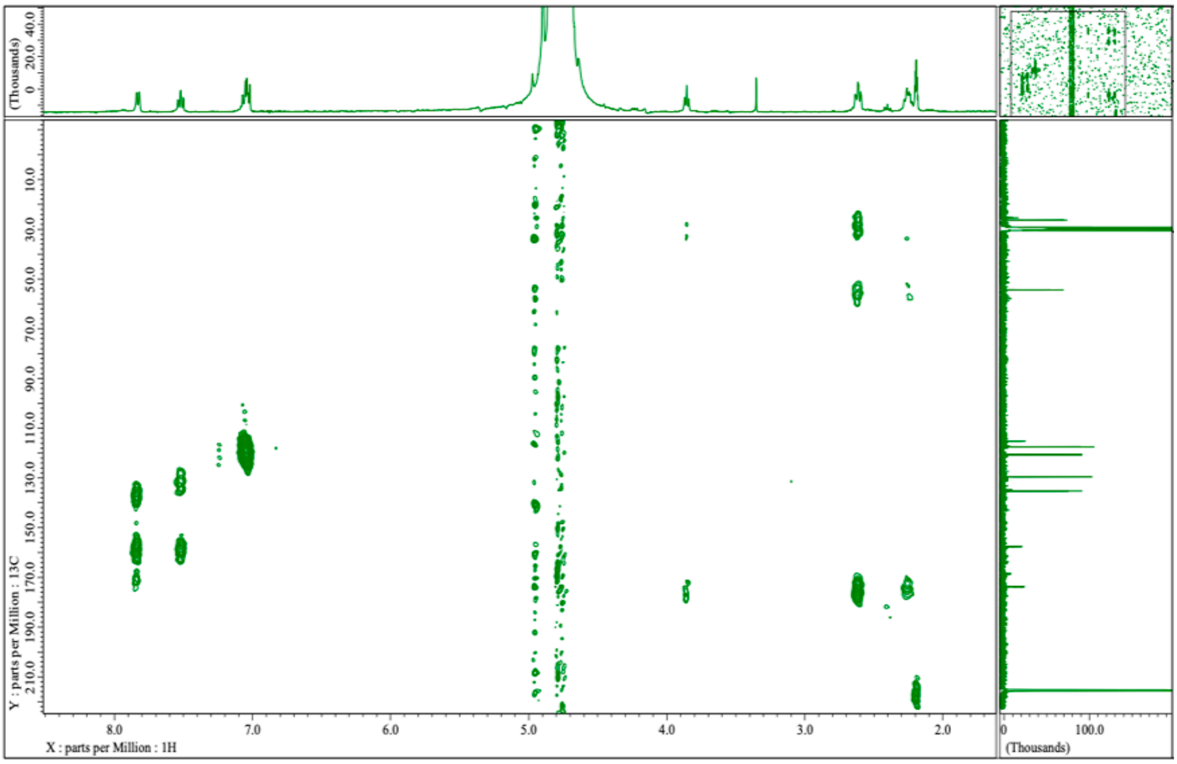

Figure S30: HMBC spectrum of **RA-Sali**

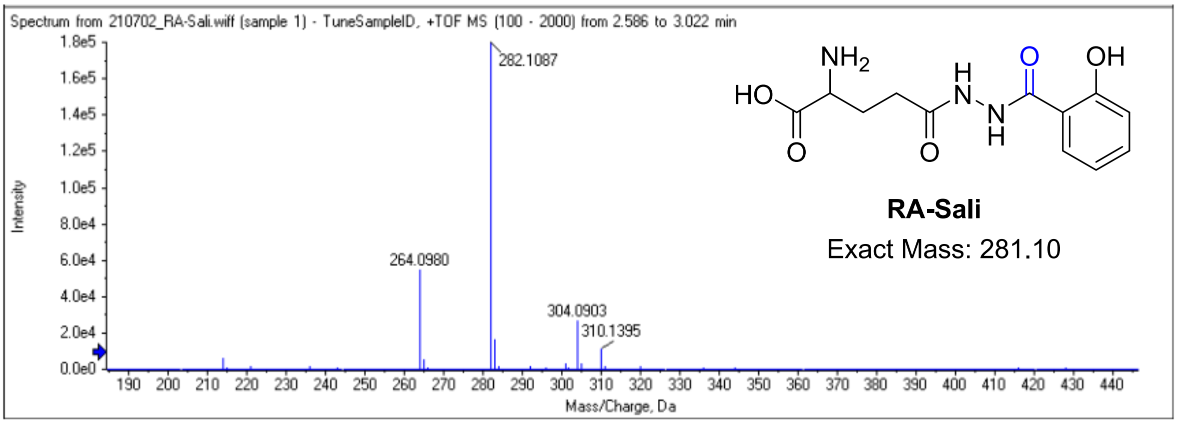

Figure S31: HRESIMS spectrum of **RA-Sali**

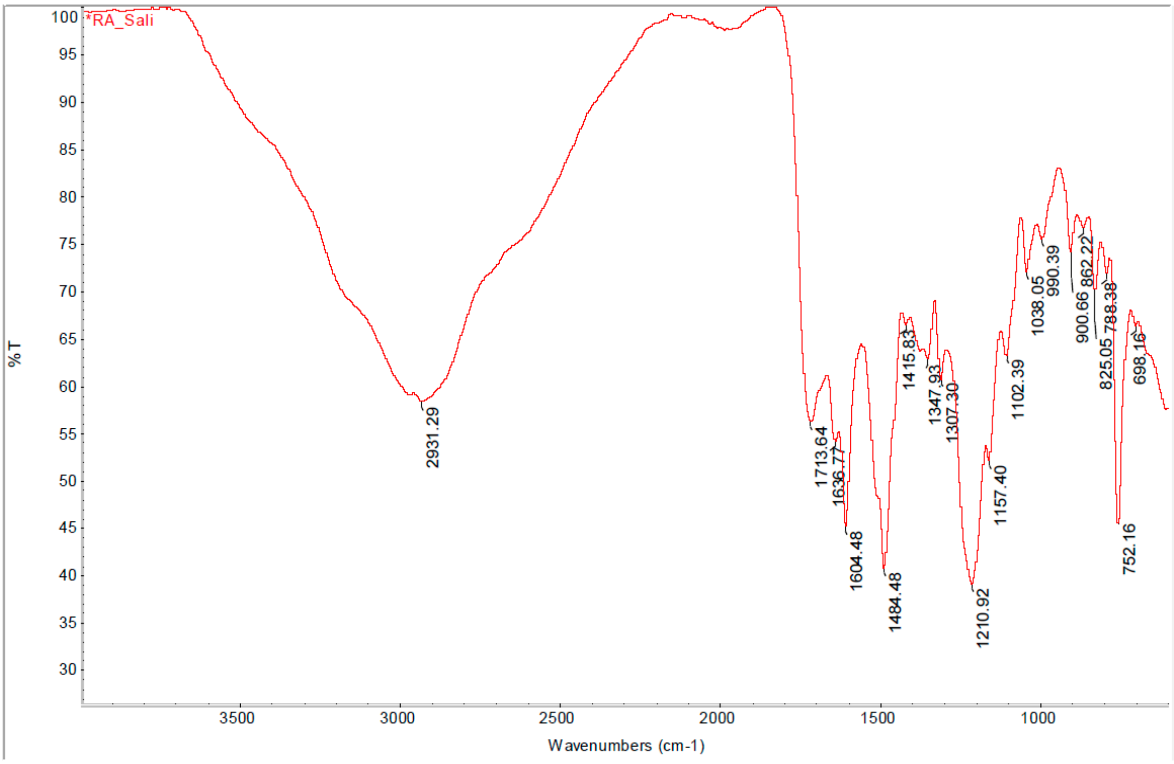

Figure S32: FT-IR spectrum of **RA-Sali**

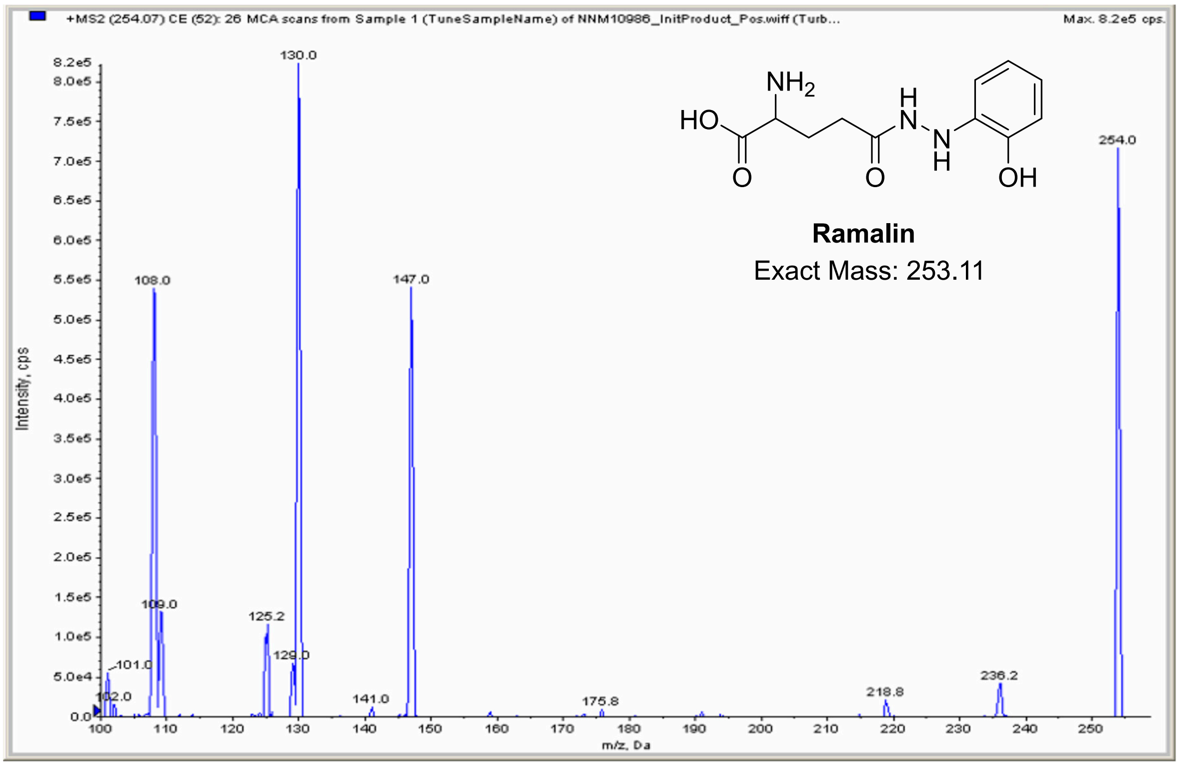

Figure S33: Mass product ion scan spectrum of **Ramalin**
